# Atypical depression is more common than melancholic in fibromyalgia: an observational cohort study

**DOI:** 10.1186/1471-2474-11-120

**Published:** 2010-06-14

**Authors:** Rebecca L Ross, Kim D Jones, Rachel L Ward, Lisa J Wood, Robert M Bennett

**Affiliations:** 1 Oregon Health & Science University, School of Nursing, 3455 SW US Veterans Hospital Road, Portland, Oregon, 97239, USA; 2 Oregon Health & Science University Cancer Institute, Division of Hematology and Medical Oncology, 3181 SW Sam Jackson Park Road, Portland, Oregon, 97239, USA; 3 Oregon Health & Science University, School of Medicine, 3181 SW Sam Jackson Park Road, Portland, Oregon, 97239, USA

## Abstract

**Background:**

It has been postulated that atypical and melancholic depression subtypes exist in depressed fibromyalgia (FM) patients, yet no study has empirically tested this hypothesis. The purpose of this study is to determine whether major depressive disorder (MDD) with atypical features and MDD with melancholic features occurs in a FM sample and to describe their demographic, clinical and diagnostic characteristics.

**Methods:**

An observational cohort study using a descriptive cross-sectional design recruited a convenience sample of 76 outpatients with FM from an academic Rheumatology clinic and a community mental health practice. Diagnoses of FM were confirmed using the 1990 ACR classification guidelines. Diagnoses of MDD and diagnostic subtypes were determined using the DSM-IV-TR criteria. Clinical characteristics were measured using the Fibromyalgia Impact Questionnaire, Structured Interview Guide for the Hamilton Depression Rating Scale with Atypical Depression Supplement and other standardized instruments. Odds ratios were computed on subtype-specific diagnostic criteria. Correlations assessed associations between subtype diagnoses and diagnostic criteria.

**Results:**

Of the 76 subjects with FM, 11.8% (n = 9) were euthymic, 52.6% (n = 40) met diagnostic criteria for MDD with atypical features and 35.6% (n = 27) for MDD with melancholic features. Groups did not differ on demographic characteristics except for gender (p = 0.01). The non-depressed and atypical groups trended toward having a longer duration of FM symptoms (18.05 yrs. ± 12.83; 20.36 yrs. ± 15.07) compared to the melancholic group (14.11 yrs. ± 8.82; p = 0.09). The two depressed groups experienced greater severity on all clinical features compared to the non-depressed group. The atypical group did not differ clinically from the melancholic group except the latter experienced greater depression severity (p = 0.001). The atypical group demonstrated the highest prevalence and correlations with atypical-specific diagnostic criteria: (e.g., weight gain/ increased appetite: OR = 3.5, p = 0.02), as did the melancholic group for melancholic-specific criteria: (e.g., anhedonia: OR = 20, p < 0.001).

**Conclusion:**

Depressed fibromyalgia patients commonly experience both atypical and melancholic depressive features; however, in this study, atypical depression was 1.5 times more common than melancholic depression. This finding may have significant research and clinical implications.

## Background

Fibromyalgia (FM) is a common, chronic and often debilitating rheumatologic disorder which presents in 7% of all primary care visits [1]. FM is characterized by widespread musculoskeletal pain (100%), specified tender points (100%), fatigue (96-100%), sleep disturbances (86%-98%), and co-morbid mood disorders (29%) [2]. While the lifetime prevalence rate of major depressive disorders in the general population is 10% to 25% for women and from 5% to 12% for men [3], 74% of FM patients report at least one major depressive disorder (MDD) episode in their lifetime [4]. Depression is known to worsen FM symptom severity including pain, functional impairment, sleep quality and quality of life [5-7]. Of grave concern is suicide is the leading cause of premature mortality in those with FM. People with FM have been reported to have a nine-fold increased risk of death from suicide during depressive episodes [8]. It is important to further elucidate the biological and psychological underpinnings of depression in FM, as the successful treatment of depression has been shown to significantly improve FM symptomatology [9].

While several subtypes of MDD have been discussed in the literature, only three have specific diagnostic criteria defined in the Diagnostic and Statistical Manual, 4 ^th ^edition, Text Revised (DSM-IV-TR) [10]: MDD with psychotic features, MDD with atypical features and MDD with melancholic features. Full diagnostic criteria can be viewed in an additional file (#1).Combined, the atypical and melancholic subtypes represent approximately 60% of all MDD cases [11] and have been postulated to represent the two main subtypes of depression in FM [12], thus are the focus of this study.

Major depressive disorder, with atypical and melancholic features, has been methodically investigated in depressed populations: yet there is still some debate as to the validity of the subtype-specific criteria, especially for atypical depression [13]. While several researchers have critiqued the current DSM-IV-TR criteria, as yet a viable alternative has not been agreed upon [14]. Therefore, for the purpose of this study, the current prevailing criteria as per the DSM-IV-TR were used. Gold and Chrousos [15] postulated that atypical depression would be more prevalent in FM patients compared to melancholic depression due to the shared biological underpinnings of atypical depression and FM. Specifically, there is a blunting of hypothalamic-pituitary-adrenal (HPA) axis functioning as evidenced by low to normal levels of plasma cortisol following dexamethasone suppression testing. This blunting is secondary to glucocorticoid receptor desensitization as a result of chronic over secretion of cortisol. However, there are no studies to date that have evaluated Gold and Chrousos' assertions. Thus the first aim of this study was to test the hypotheses that atypical depressive episodes (ADE) and melancholic depressive episodes (MDE) occur in FM patients with ADE being the predominant subgroup. A secondary aim was to describe the demographic and clinical characteristics and diagnostic features of depression subgroups in people with FM to determine if they exhibited the same symptom clusters as those in depressed, non-FM samples.

## Methods

### Sample and data collection

Subjects were recruited from an academic medical center in the Pacific Northwest and a local community mental health clinic. A multi-step recruitment protocol was used to invite subjects to enter the study [16]. Males and females 18 years old or older who were diagnosed with FM as per the 1990 American College of Rheumatology criteria [17] for ≥ 2 years were eligible to participate in the study. Subjects also needed to speak and read English at a 6 ^th ^grade level. Exclusion criteria, designed to decrease risk to subjects and potential confounding variables, excluded subjects who were acutely ill, pregnant, currently lactating or planning to conceive within 90 days. Additional exclusion criteria included a Beck Depression Inventory score greater than 31 (extreme depression), any medical disorder that altered the HPA axis, suicidal ideation, abnormal thyroid stimulating hormone levels (less than 0.28 uIU/ml or greater than 5.00 uIU/ml), weight change greater than 15 pounds during the prior three months and subjects who did not meet diagnostic criteria for one of the three FM groups, i.e.: non-ADE or non-MDE (n = 1). Subjects on medications that could potentially alter the HPA axis were also excluded (e.g.: prednisone, opioids, corticosteroids, carbamazepine, etc.). Those on anti-depressants were not excluded, as research has shown the presence or absence of antidepressants while depressed, type of antidepressant or number of antidepressant trials previously used did not affect HPA axis perturbations or cortisol levels (unless depression symptoms had resolved) following the combined dexamethasone suppression/ corticotrophin-releasing hormone (DEX/CRH) test [18]. Thus depressed subject's plasma cortisol levels, which are associated with subtype-specific symptom expression, would still be expected to reflect MDD subtype variations. Approval of the protocol was obtained from the university's Investigational Review Board. Data was collected from 01/07/2006 to 10/18/2006.

### Protocol

The first visit was held at the University's School of Nursing in a town hall meeting format. All subjects gave written informed consent after an explanation of the study's purpose and procedures was given. Screening information for depression, demographic data, current medications and medical history were obtained and inclusion/exclusion criteria were assessed. In an attempt to decrease the possibility of estrogen fluctuations affecting symptom presentation, (e.g., fatigue, amotivation, hyperphagia and hypersomnia), menstruating females who continued to meet criteria were encouraged to schedule and attend the second visit during their next luteal phase (14 days after the first day of their last menstrual cycle), when estrogen levels would theoretically be at their highest. Menstrual phase was determined from subjective reports of the number of days from the first day of the last menstrual period. All other subjects were scheduled within 30 days of their first assessment.

At visit 2, subjects completed self-report instruments plus interviewer-administered evaluations. To decrease inter-rater reliability issues, the principal investigator (PI) completed all physical examinations and interviewer-administered questionnaires for all subjects. The following instruments, chosen specifically for their psychometric properties and appropriateness for use in an FM population, were used to assess depression severity, subtype-specific diagnostic criteria, overall FM symptom severity, pain severity, functional impairment, quality of life and sleep quality.

### Primary measures

The 2003 version of the *Structured Interview Guide for the Hamilton Depression Rating Scale with Atypical Depression Supplement (SIGH-ADS) *was used to assess overall depression severity and severity of subtype-specific diagnostic features. This interviewer-administered questionnaire is based on the 21-item Hamilton Depression Rating Scale (HAM-D) plus includes an 8-item addendum to assess atypical depressive episode (ADE) features [19]: social withdrawal, increased appetite, increased eating, weight gain, carbohydrate craving or eating, hypersomnia, fatigability and pattern of depression symptoms being worse in the afternoon. It also includes two un-scored questions regarding difficulty awakening and temperature discomfort that are indicative of ADE. The HAM-D also includes seven items that assess features of melancholic depressive episode (MDE) features as per the DSM-IV-TR [10]: loss of appetite, weight loss, terminal insomnia, guilt, agitation, psychomotor retardation and pattern of depression symptoms being worse in the morning. Scores range from 0 to 88 with lower scores reflective of lower depression severity. Reliability for scale items of the SIGH-ADS as measured by Cronbach's alpha was established to be 0.87 in this sample.

To insure instrumental validity of the SIGH-ADS, a patient self report version was used to verify the interviewer-obtained responses. The *Structured Interview Guide for the Hamilton Depression Rating Scale, Seasonal Affective Disorder- Self Report Version (SIGH-SAD-SR)*[20] includes the same questions as the SIGH-ADS and has the same scoring matrix. After the SIGH-ADS was administered, the self-report questionnaire was reviewed by the PI. If responses differed by two or more points on any question, the PI clarified the question with the subject and rescored it accordingly. Reliability for scale items of the SIGH-SAD-SR as measured by Cronbach's alpha was established to be 0.78 in this sample.

The DSM-IV-TR diagnostic criteria for MDD with atypical features and MDD with melancholic features [10] were used to diagnose depressive subtypes. Based on these criteria, subjects were divided into three groups: A. non-depressed FM patients, B. FM patients with a current ADE and C. FM patients with a current MDE. As only one depressed FM subject did not meet diagnostic criteria for either ADE or MDE (non-ADE/non-MDE, n = 1), this data could not be used for group comparisons, thus was excluded from all statistical analyses.

The *Fibromyalgia Impact Questionnaire*(FIQ) was used to measure clinical features of FM, including pain severity, functional impairment, fatigue and depression. The FIQ was developed to measure the components of health status believed to be most affected by FM symptom severity, impact on functionality and response to treatment and outcomes in interventional studies [21]. Total FIQ scores range from 0 to 100, with higher values indicating a more negative impact of FM. The test-retest reliability has been documented to range from 0.56 on the pain score to 0.95 for physical function [22]. Internal consistency as measured by Cronbach's alpha has been established to range from 0.72 to 0.93 in seven translated versions of the FIQ. Reliability for scale items of the FIQ as measured by Cronbach's alpha was established to be 0.85 in this sample.

Three instruments were used to measure pain dimensions: the *FIQ *visual analogue scale (VAS) for pain, the number of *tender points *and the *cumulative myalgic score *(CMS). The 100-mm VAS for pain rated the participant's perception of pain intensity over the previous 7 days. The CMS is an eighteen-item scale that diagnoses FM as per the 1990 ACR criteria plus rates the amount of pain associated with 4-kilograms of pressure applied to 18 tender points commonly found in FM. The number of non-zero scores out of 18 determined the total tender point count. Tenderness and pain severity were measured by observing subject's reactions to 4-kg pressure on a 0-3 scale (0 = no pain, 1 = some pain, 2 = verbal exclamation (e.g. "ouch"), 3 = flinches/ moves away). Higher scores indicate more pain, with a total possible range of 0 to 54. The PI performed all tender point evaluations. Reliability for scale items of the CMS as measured by Cronbach's alpha was established to be 0.90 in this sample.

The *Flannigan Quality of Life Scale *(QOLS) is a16-item Likert-type scale that assesses multiple areas of well-being and life satisfaction [23]. Quality of life is measured on a continuum where 1 = terrible and 7 = delighted. The possible range of scores is from 16 to 112, with higher scores indicating better well-being and quality of life. It has been validated in an FM sample with an internal consistency reliability alpha equaling 0.82 to 0.88 and test-retest reliability of 0.84. Reliability for scale items of the QOLS as measured by Cronbach's alpha was established to be 0.92 in this sample.

The *Jenkins Sleep Scale *was used to assess sleep quality. It is a 4-item scale divided over 5 equal time periods during the preceding month that measures the quality of sleep obtained [24].The scale has a possible range of scores from 0 to 20, with higher scores indicating poorer overall quality of sleep. The internal consistency coefficient for the scale was 0.79. Reliability for scale items of the Jenkins Sleep Scale as measured by Cronbach's alpha was established to be 0.80 in this sample.

### Secondary measures

The *Demographic Data Form*, an investigator-designed questionnaire, was used to screen for inclusion/exclusion criteria (past medical history, review of systems, current medications) and to obtain demographic information including age, gender, ethnicity/race; educational level; marital and employment status; number of years with FM; and body mass index (BMI). Body weight and height were measured in kilograms and meters using a calibrated standing model scale (Detecto, Brooklyn, New York). A standard formula of weight in kilograms divided by height in meters squared was used to calculate BMI.

The *Beck Depression Inventory-II-1973 Revision *(BDI-II-R) was used to screen for eligibility based on overall depression severity and confirm the diagnosis of MDD. The BDI-II-R is a 21-item scale that assesses intensity of depression in clinical patients and in healthy controls and has a reliability of 0.92 [25]. It has been widely used in depression research and has been adapted for use in FM by removing three items from the total score: fatigue, sleep disturbance, and effort to get things done. These symptoms correspond to FM symptomatology and therefore do not correlate well with MDD and overestimate the level of depression. This adaptation, the BDI-A, has better sensitivity (74% - 85%) and specificity (45% - 65%) in a FM population than the original [26]. A score of 13 or higher indicates a moderate level of depression, while scores above 21 are fairly specific for MDD in FM patients [26]. Reliability for scale items of the BDI-A was determined to be a Cronbach's alpha of 0.87 in this sample.

### Statistical analyses

Data analyses were conducted using the Statistical Package for Social Sciences version 15.0 (SPSS, Chicago, Illinois, USA). Significance levels were set at an alpha of ≤ 0.05 and used a 2-tail distribution. Chi-square (χ^2^) tests were used to determine if there were differences between groups on nominal and ordinal level variables and also to determine if diagnostic groups differed statistically on prevalence of subtype-specific diagnostic criteria. To measure the differences among diagnostic groups on interval level variables, analysis of variance (ANOVA) with Bonferroni post-hoc tests were conducted with an adjusted alpha of 0.01 for multiple comparisons. To assess the association between depression subtypes and diagnostic criteria, multivariate analyses using logistic regression with odds ratios were used. Relationships between each criterion and each MDD subtype (ADE vs. MDE) were further examined using Spearman's Rho correlation coefficients.

## Results

### Demographic characteristics

**Figure 1:**
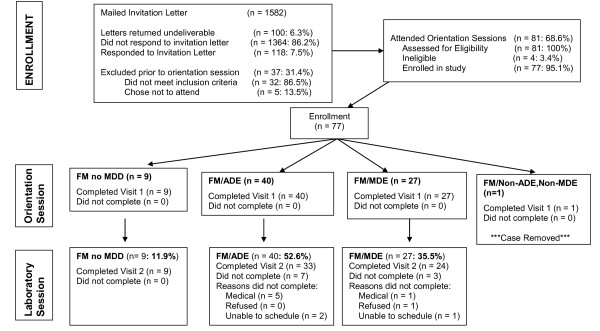
Enrollment Flowchart

Convenience sampling identified 118 potential subjects who were assessed for eligibility. Of those, 95% (n = 77/118) met inclusion criteria, signed an informed consent and completed Visit 1. One subject's data was later excluded due to not meeting depression subtype group inclusion criteria; thus the total sample consisted of 76 subjects. Ten subjects were unable to complete Visit 2; therefore analyses for clinical characteristics were conducted on the 66 subjects who completed all evaluations. See Figure 1 for full recruitment and enrollment details. All subjects (n= 63) were tested within an average of 17 (SD ± 20.43) days from the first assessment. Both premenopausal and post-menopausal females were seen an average of 14 days after their first assessment (Premenopausal: SD ± 12.82; Postmenopausal: SD ± 14.53).

Subjects were on average 54 years old (± 12.7 yrs.) and had experienced FM symptoms for an average of 18 years-Table 1. Nine of the total sample of 76 FM subjects (11.8%) did not meet diagnostic criteria for MDD, 40 (52.6%) met criteria for ADE, and 27 (35.6%) for MDE. The total sample reflected the expected predominance of females (96%) versus males (4%) [27]. No significant differences existed between groups on demographic variables except on gender (p = 0.01). Symptom duration approached significance (p = 0.09) with the ADE subgroup reporting FM symptoms an average of 6 years longer (20 years ± 15.1) than the MDE subgroup (14 years ± 8.8). The duration of symptoms in the non-MDD group, (18 years ± 12.8), did not differ statistically from the ADE group.

**Table 1:** Comparison of demographic data by diagnostic group: count (%), mean (±SD) and *X*^2 ^values

DEMOGRAPHIC VARIABLES		Total Sample	No MDD	ADE	MDE	*BTW Group*
		(n = 76) 100%	(n = 9) 11.8%	(n = 40) 52.6%	(n = 27) 35.6%	*P Values*
**Gender**	Female	73 (96.1%)	7 (77.8%)	39 (97%)	27 (100%)	P = .01
	Male	**3 (3.9%)**	2 (22.2%)	1 (2.5%)	0	

**Ethnicity**	Hispanic/Latino	**5 (6.6%)**	0 (0%)	3 (7.5%)	2 (7.4%)	P = .81
	Non-Hispanic/ Non-Latino	**67 (88.2%)**	8 (88.9%)	35 (87.5%)	24 (88.9%)	
	Not reported/Unknown	**4 (5.3%)**	1 (11.1%)	2 (5.0%)	1 (3.7%)	

**Race**	Asian	**1 (1.3%)**	0	1 (2.5%)	0	P = .16
	Black/African American	**2 (2.6%)**	0	0	2 (7.4%)	
	American Indian/ Alaskan Native	**5 (6.6%)**	2 (22.2%)	1 (2.5%)	2 (7.4%)	
	White	**68 (89.5%)**	7 (77.8%)	38 (95%)	23 (85.2%)	

**Educational Level**	Grade 10-12	**6 (7.9%)**	0	3 (7.5%)	3 (11.1%)	P = .47
	High School Diploma/GED	**9 (11.8%)**	0	6 (15%)	3 (11.1%)	
	Some college or trade school	**33 (43.4%)**	6 (66.7%)	14 (35%)	13 (48.2%)	
	College Degree or Higher	**28 (36.8%)**	3 (33.3%)	17 (44.5%)	8 (29.6%)	

**Marital Status**	Single/Separated	**25 (32.9%)**	0	12 (30%)	13 (48.1%)	P = .14
	Married/Living Together	**47 (61.8%)**	9 (100%)	26 (65%)	12 (44.5%)	
	Other	**4 (5.3%)**	0	2 (5%)	2 (7.4%)	

**Employment Status**	Full Time	**16 (21.1%)**	3 (33.3%)	8 (20%)	5 (18.5%)	P = .70
	Part Time	**8 (10.5%)**	0	4 (10%)	4 (14.8%)	
	Not employed out of home	**52 (68.4%)**	6 (66.7%)	28 (70%)	18 (66.7%)	

**Receiving disability**	No	**53 (69.7%)**	7 (77.8%)	27 (67.5%)	19 (70.4%)	P = .83
	Yes	**23 (30.3%)**	2 (22.2%)	13 (32.5%)	8 (29.6%)	

**Age in years**		**54.36 (12.67)**	52.22 (11.16)	55.20 (12.95)	53.81 (13.05)	P = .79

**# of yrs with FM symptoms**		**18.05 (12.83)**	20.11 (10.78)	20.36 (15.07)	14.11 (8.82)	P = .09

**# of years diagnosed with FM**		**7.72 (6.12)**	10.22 (8.20)	7.40 (6.10)	7.35 (5.36)	P = .43

**BMI**		**30.55 (19.69)**	29.51 (6.97)	32.05 (7.35)	29.08 (6.57)	P = .28

Chi-square computed for nominal and ordinal data; ANOVA computed for interval data.

### Clinical characteristics

As depicted in Table 2, groups differed on all clinical characteristics except for number of tender points and stiffness. All significant analyses of clinical features remained significant at an adjusted Bonferroni alpha level of 0.01 with the exceptions of physical impairment (p = 0.02) and pain severity as measured by the cumulative myalgic score (p = 0.02). Pain as evidenced by the FIQ VAS was highly significant (p < 0.001). Irrespective of subtype, non-depressed subjects were generally less symptomatic as measured by the total FIQ score (non-MDD vs. ADE: p < 0.001; non-MDD vs. MDE: p < 0.001), had a better quality of life (non-MDD vs. ADE: p < 0.001; non-MDD vs. MDE: p < 0.001) and quality of sleep (non-MDD vs. ADE: p = 0.007; non-MDD vs. MDE: p = 0.012) compared to subjects with MDD.

**Table 2:** Comparison of clinical characteristics for total sample and by diagnostic group: mean (± SD)

Clinical Characteristics	No MDD	ADE	MDE	P value	Between Group
Construct (Instrument)	(n = 9)	(n = 33)	(n = 24)		Significance
	A	B	C		
**Pain-Presence**	16.33 (2.50)	17.55 (1.00)	17.63 (.82)	0.60	NS
(Number of tender points)					

**Pain-Severity**					a-b*
(Cumulative Myalgic Score)	34.67 (11.70)	43.72 (8.29)	44.42 (8.65)	0.02	a-c*

**Pain-Tenderness**	4.11 (2.52)	6.58 (1.87)	7.54 (1.74)	< 0.001	a-b*
(FIQ VAS)					a-c*

**Depression Severity**	4.89 (3.72)	16.70 (5.78)	22.25 (3.93)	< 0.001	a-b***
(HamD-17)					a-c***
					b-c***

**Total FIQ Score **	31.45 (18.57)	64.04 (17.72)	68.70 (10.75)	< 0.001	a-b***
					a-c***

**Depression Severity **	1.11 (1.67)	6.03 (5.51)	6.71 (2.49)	< 0.001	a-b***
(FIQ VAS)					a-c***

**Stiffness**	5.71 (4.27)	7.23 (2.28)	7.15 (2.58)	0.26	NS
(FIQ VAS)					

**Physical Impairment**	2.98 (2.17)	4.89 (2.22)	5.32 (1.73)	0.02	a-b*
(FIQ)					a-c*

**Quality of life**	95.56 (8.23)	69.58 (15.72)	63.83 (15.71)	< 0.001	a-b***
(Flannigan)					a-c***

**Quality of sleep**				0.006	a-b**
(Jenkins)	7.89 (7.52)	14.15 (4.78)	14.00 (4.78)		a-c**

ANOVA computed for interval data. * P ≤ 0.05, ** P ≤ 0.01, *** P ≤ 0.001.

Comparisons of the ADE subgroup versus the MDE subgroup revealed depression severity was significantly higher in the MDE group compared to the ADE group as measured by the HamD-17 embedded in the SIGH-ADS (p < 0.001). No significant differences in overall impact of FM symptoms on daily life (p = 0.44), quality of life (p = 0.49), sleep quality (p = 1.0), pain severity (p = 0.2), or tenderness (p = 1.0) existed between groups.

### Subtype-specific criteria

As expected, the prevalence rates of the subtype-specific diagnostic criteria for MDD with melancholic features as per the DSM-IV-TR (Table 3) were found to be highest in the MDE group and the criteria for MDD with atypical features (Table 4) were highest in the ADE group.

**Table 3:** Prevalence rates of melancholic depressive episode-specific diagnostic criteria by diagnostic group

MDE CRITERIA	No MDD	ADE	*MDE*	**χ**^**2**^	ADE v. MDE (n = 67)	
	(A: n = 9)	(B: n = 40)	*(C: n = 27)*	P value	OR	*P *Value
Anhedonia	0/9	5/39	***7/27 ***	**TS < 0.001**	20.0	< 0.001
(loss of pleasure in all,	0%	12.5%	***74.1%***	a-b 0.57		
or almost all activities)				**a-c < 0.001**		
				**b-c < 0.001**		

Lack of reactivity to	1/9	0/39	***8/27***	**TS < 0.001**	1.0	
usually pleasurable stimuli	11.1%	0%	***29.6%***	a-b 0.19	(Perfect Predictability)	
				a-c 0.40		
				**b-c < 0.001**		

Distinct quality of	0/9	34/40	***27/27***	**TS < 0.001**	1.0	
depressed mood	0%	85%	***100 %***	**a-b < 0.001**	(Perfect Predictability)	
				**a-c < 0.001**		
				b-c 0.07		

Depression regularly	1/9	13/41	***17/27***	**TS < 0.007**	3.5	0.02
worse in the morning	11.1%	32.5%	***63%***	a-b 0.41		
				**a-c 0.02**		
				**b-c 0.02**		

Early morning awakening	3/9	17/40	***24/27***	**TS < 0.001**	10.8	< 0.001
	33.3%	42.5%	***88.9%***	a-b 0.72		
				**a-c 0.003**		
				**b-c < 0.001**		

Marked psychomotor	0/9	13/40	***19/27***	**TS < 0.001**	4.9	0.003
retardation or agitation	0%	32.5%	***70.4%***	a-b 0.09		
				**a-c < 0.001**		
				**b-c 0.003**		

Significant anorexia or	1/9	3/40	***9/27***	**TS = 0.02**	6.2	0.01
Weight loss	11.1%	7.5%	***33.3%***	a-b 0.57		
				a-c 0.39		
				**b-c 0.007**		

Excessive or	0/9	16/40	***23/27***	**TS 0.001**	8.6	< 0.001
inappropriate guilt	0%	40%	***85.2%***	**a-b 0.02**		
				**a-c < 0.001**		
				**b-c < 0.001**		

**Abbreviations**: TS, Total Sample; CI, Confidence Interval; OR, odds ratio. Italicized cells reflect highest prevalence rates among the 3 diagnostic groups.

**Table 4:** Prevalence rates of atypical depressive episode-specific diagnostic criteria by diagnostic group

ADE CRITERIA	No MDD	*ADE*	MDE	**χ**^**2**^	ADE v. MDE (n = 67)	
	(A: n = 9)	*(B: n = 40)*	(C: n = 27)	P value	OR	*P *Value
Mood reactivity	4/9	***40/40***	20/27	**TS < 0.001**	1.0	
	44.4%	***100%***	74.1%	**a-b < 0.001**	(Perfect Predictability)	
				a-c 0.13		
				**b-c 0.001**		

Significant weight gain or	0/9	***27/40***	10/27	**TS < 0.001**	3.53	0.02
increased appetite	0%	***67.5%***	37%	**a-b < 0.001**		
				**a-c 0.04**		
				**b-c 0.02**		

Hypersomnia	0/9	***14/40***	5/27	TS 0.06	2.37	0.15
	0%	***35%***	18.5%	a-b 0.05		
				a-c 0.30		
				b-c 0.18		

Leaden paralysis (i.e., heavy,	4/9	***40/40***	26/27	**TS < 0.001**	1.0	
leaden feelings in arms or legs)	44.4%	***100%***	96.3%	**a-b < 0.001**	(Perfect Predictability)	
				**a-c 0.002**		
				b-c 0.40		

Long-standing pattern of	0/9	***27/38***	13/27	**TS < 0.001**	2.64	0.06
interpersonal rejection sensitivity	0%	***71.1%***	48.1%	**a-b < 0.001**		
				**a-c 0.01**		
				b-c 0.07		

Criteria not met for MDE during	9/9	***40/40***	0/27	**TS < 0.001**	0.007	0.007
the same episode	100%	***100%***	0%	a-b 1.00		
				**a-c < 0.001**		
				**b-c < 0.001**		

**Abbreviations**: TS, Total Sample; CI, Confidence Interval; OR, odds ratio. ***Italicized ***cells reflect highest prevalence rates among the 3 diagnostic groups.

#### Atypical Depressive Episode-specific criteria

While groups exhibited predominant symptom patterns respective of their diagnostic classification (ADE vs. MDE), some individual symptoms overlapped between groups. In our sample, all subjects in the ADE group reported *mood reactivity*, a mandatory criterion for ADE, while 74.1% of the MDE and 44.4% of the non-MDD groups also experienced this symptom. Over 67% of the ADE group experienced weight gain or increased appetite. Interestingly, 37% of the MDE group also reported weight gain, while only 33% reported significant anorexia or weight loss, which is the MDE-specific criterion. The symptom of *hypersomnia *was highest in the ADE group (35%) followed by the MDE group (18.5%). Over 71% of the ADE group and 48.1% of the MDE group reported *interpersonal rejection sensitivity*. Of interest, the majority of the total sample reported *leaden paralysis *irrespective of diagnostic subgroup, with 100% of the ADE group, 96.3% of the MDE group and 44.4% of the non-MDD group reporting this ADE-specific criterion. Groups differed significantly on chronicity of depression as measured by the number of episodes of depression [χ^2 ^(2, n = 51) = 67.84, p = 0.007], with the ADE group having the most episodes. Although groups did not differ on the number of times suicide was attempted [χ^2 ^(2, n = 61) = 4.16, p = 0.13], the highest prevalence of attempts were made by the ADE group (38.1%; n = 8/21), followed by the MDE group (15%; n = 3/20). None of the non-depressed group had ever attempted suicide.

#### Melancholic Depressive Episode-specific criteria

The prevalence rate of *anhedonia *was 74% in the MDE group while 12.5% of the ADE group also met this MDE-specific criterion. Over 29% of the MDE group and 11.1% of the ADE group reported *lack of reactivity to usually pleasurable stimuli*. A *distinct quality of depressed mood *was present in 100% of the MDE group; but 85% of the ADE group also experienced this symptom. *Depression *was *regularly worse in the morning *in 63% of the MDE group and 32.5% of the ADE group. *Early morning awakening *was highest in the MDE group (88.9%), but almost half of the ADE group and over a third of the non-MDD group also reported this symptom. *Marked psychomotor retardation or agitation *was highest in the MDE group (70.4%), yet 32.5% of the ADE group also endorsed it. *Excessive or inappropriate guilt *was highest in the MDE group (85.2%), compared to 40% in the ADE group.

### Odds ratios

The presence of the MDE-specific criteria of *lack of reactivity to usually pleasurable stimuli *and *distinct quality of depressed mood *predicted the diagnosis of MDE. Likewise, leaden paralysis and mood reactivity predicted the diagnosis of ADE. However, while the associations of the diagnostic subtypes with the above features were very sensitive (the diagnosis of ADE was associated 100% of the time with leaden paralysis) it was not very specific (leaden paralysis was associated with the diagnosis of MDE 47% of the time). As odds ratios cannot be computed on items that perfectly predict each other, the above four criteria were removed from further analyses.

All other odds ratios were positive, indicating subtype-specific diagnostic criterion were highly associated with their respective subtype. As seen in Table 3, the MDE group was 20 times more likely to endorse anhedonia (p < 0.001); 3.5 times more likely to endorse depression being worse in the mornings (p < 0.02); 10.8 times more likely to endorse early morning awakening (p = 0.001); 4.9 times more likely to endorse psychomotor agitation or retardation (p = 0.003); 6.2 times more likely to endorse anorexia or weight loss, (p = 0.01); and 8.6 times more likely to endorse excessive or inappropriate guilt (p = 0.001) than the ADE group. In contrast, Table 4 shows the ADE group was more likely to endorse the ADE-specific criteria of weight gain/ increased appetite (OR = 3.5, p = 0.02) and interpersonal rejection sensitivity (OR = 2.6, p = 0.06) compared to the MDE group.

### Correlations

The correlation matrix of the diagnosis of ADE versus MDE with all five of the ADE-specific diagnostic features and eight MDE-specific diagnostic features can be viewed in an additional file (#2). In brief, there was a weak relationship between the diagnosis of ADE and significant weight gain/ hyperphagia (r = 0.30, p = 0.013) and a moderate relationship with mood reactivity (r = .42, p < 0.001). However, no significant associations existed between the diagnosis of ADE and hypersomnia (r = .18, p = 0.15), leaden paralysis (r = .15, p = 0.23) nor interpersonal rejection sensitivity (r = .23, p = 0.06).

For the MDE-diagnosed group, there was a strong relationship between the MDE-specific criteria anhedonia (loss of pleasure in all, or almost all, activities) (r = 0.62, p < 0.001), and moderate relationships with lack of reactivity to usually pleasurable stimuli (r = 0.45, p < 0.001), early morning awakening (r = 0.47, p < 0.001) and excessive or inappropriate guilt (r = 0.45, p = < 0.001). Weak relationships existed with marked psychomotor retardation or agitation (r = 0.37, p = 0.002), significant anorexia or weight loss (r = 0.33, p = 0.006), depression worse in the morning (r = 0.30, p = 0.013) and distinct quality of depressed mood (r = 0.26, p = 0.035).

## Discussion

The results reported herein provide evidence for the occurrence of atypical and melancholic subtypes of depression in FM subjects. Furthermore, these subjects exhibited similar clinical features of ADE and MDE as has been reported in other depressed non-FM populations [13,28,29]. Interestingly, the ADE prevalence rate of 52.6% is approximately twice that of the 30% reported in population studies of depressed people without FM. This finding is consistent with ADE being more prevalent in women and may be associated with the neuroendocrine underpinnings of the preponderance of women versus men (9:1) with FM [30]. The prevalence rate of MDE was 35.6%, which is more consistent with previous studies in depressed non-FM populations which demonstrated a prevalence of approximately 30% [31,32]. Demarcation of MDD subtypes is a novel area of research in FM. Prior to completing this pilot study, it was not known if subtype prevalence in an FM sample would be consistent with the prevalence of MDD subtypes previously identified in the general population. We speculate the over-representation of ADE is likely due to the above and ADE being more prevalent in females in addition to the long-term effect of chronic stress blunting HPA axis functions resulting in lower cortisol levels. It is unclear as to why only one subject out of 77 was identified with the non-ADE/non-MDE subtype. It is possible this finding was associated with self selection bias, differences in symptom presentation of MDD co-morbid with FM versus MDD alone, or the possibility the subject was transitioning from MDE to ADE. A larger study is needed to better characterize depression subtypes and their clinical features to determine if the above finding is reproducible. Our team plans to investigate this issue in future studies.

### Clinical features of FM more severe in depressed groups

Our data substantiates previous reports that associate a diagnosis of MDD in FM patients with more pain [33], poorer sleep quality [34], poorer quality of life [35] and greater functional impairment [36]. However, in the current study, the specific subtype of MDD (ADE vs. MDE) did not affect the intensity of the clinical characteristics of FM with the exception of greater depression severity in the MDE group.

### Overlap of diagnostic criteria between subtypes

While conceptually, MDD subtype-specific criteria are exclusive to their respective diagnostic subtype with no overlap between subgroups, ours and other study data [13,37] indicate diagnostic features that differentiate ADE versus MDE do commonly overlap. Benazzi provides an in-depth discussion of the concept of depressive subtype symptom overlap in depressed non-FM populations and presents the hypothesis of mood disorders representing a continuum of overlapping disorders with common underlying biological pathways versus being absolute categorical definitions [38]. Consistent with our findings, Angst and colleagues [39] found in their 20 year prospective study that depressed non-FM subjects with pure melancholic and atypical depression exhibited many similar characteristics, as evidenced by no significant differences between groups on psychomotor retardation/agitation, weight gain or feelings of excessive or inappropriate guilt. In a large trial of 579 depressed non-FM patients (ADE, n = 130; Non-ADE, n = 449) ADE symptoms were reported in both groups, with the ADE group having the highest prevalence of mood reactivity (100% vs. 77.7%), hypersomnia (36.2% vs. 16.8%), hyperphagia (53.1% vs. 21.8%), leaden paralysis (60.8% vs. 28%), and interpersonal rejection sensitivity (75.4% vs. 40.9%) [13]. In line with Thase and colleagues [40], we found that *mood reactivity*, a mandatory criterion for ADE, was reported by the majority of the MDE group and almost half of the non-MDD group; which is also congruent with the observations of Henkel and colleagues [41]. We cautiously speculate the overlap in depressive subtype symptoms is likely due at least in part to dysfunction of the HPA axis as well as other neuroendocrine (e.g., serotonin and norepinephrine) and neuroimmune (e.g., pro-inflammatory cytokine response) systems. Although Juruena and Cleare [42] reviewed symptom overlap between ADE and other MDD subtypes in populations similar to FM including chronic fatigue syndrome, to our knowledge this study was the first to evaluate ADE and MDE specific diagnostic criterion in an FM population. Therefore, these findings need to be replicated in a larger sample before any conclusive assumption regarding the causes of the symptom overlap can occur.

One final example of the non-specificity of diagnostic criteria in our study was the finding that 100% of the ADE group endorsed experiencing "leaden paralysis"; while three-fourths of the MDE group also experienced this symptom. As the reported prevalence of leaden paralysis is significantly higher than reports from depressed non-FM populations [37], we hypothesized there may be an etiological link between leaden paralysis and the symptom of stiffness, a common complaint of FM subjects [43]. However we only found a week association between these symptoms (r = 0.26, p = 0.03) indicating there may be a mediating or moderating variable accounting for this clinical observation.

### Possible temporal association between length of time with FM and MDD subtypes

A non-significant (p = 0.09), yet potentially interesting finding was a trend among MDD subtypes to differ in the number of years that FM symptoms were present. Similar to Wallace's findings [44], the ADE and non-MDD groups had symptoms of FM for approximately six years longer than the MDE group. It has been reported that non-FM patients with ADE have a longer duration of symptoms versus non-FM patients with MDE [39]. Gold and Chrousos [15] have hypothesized that new onset FM is generally associated with a hyperactive HPA axis characterized by elevated levels of CRH and cortisol (a characteristic of MDE), and that, over time, persistent stress results in blunting of the HPA axis response (a characteristic of ADE). Our data supports this notion, and we postulate that this finding may explain disparities in previous reports of HPA axis studies in FM [45-48], as both the duration of FM symptoms and depression subtype might be expected to influence HPA axis functioning.

### Limitations

Certain groups were excluded to decrease risk to vulnerable individuals (children, severely depressed people, people with suicidal ideation) and to eliminate potential confounding variables (persons with co-occurring rheumatic and pituitary disease, therapy with medications known to alter HPA axis function), thus result may not generalize to such individuals. Varying estrogen levels across the menstrual cycle presents a potential confounding variable in this study and necessitates more in-depth measurement via standardized biochemical assays in future studies. Furthermore, the sample was recruited from patients seen at a tertiary care facility for FM consultation and short-term management and an outpatient mental health clinic specializing in mood disorders with concurrent chronic pain, thus their symptoms may have been more severe than patients managed in primary care clinics. As the sample size was relatively small, these findings need to be confirmed in a larger sample from a more diverse population. The age of onset (before vs. after 20 years old), duration of symptoms (less than vs. greater than 2 years) and chronicity of depression (number of lifetime episodes) needs to be evaluated in future studies, as these are proposed additions to the DSM-V diagnostic criteria for depression with atypical features and may add sensitivity and specificity to the diagnosis [49].

### Future directions

There is much contemporary interest in trying to understand psycho-neuro-endocrine dysfunction in relation to the etiology of FM and chronic pain. Currently these studies have yielded non-consistent and often contradictory findings. We deduce that failure to distinguish between depressive subgroups could have led to confounding results in studies that have measured HPA activity, as the HPA axis in ADE is purported to be relatively hypo-cortisolemic compared to the HPA axis in MDE. For instance, recent research has reported that interpersonal rejection sensitivity and the associated fear of negative social evaluation is associated initially with a hypercortisolemic response consistent with melancholic depression and then later in life with hypocortisolemic states such as atypical depression [50]. Thus both the duration of a stressor and the nature of an associated mood disorder are potentially important confounding variables to consider in any HPA axis study in chronic disease.

## Conclusions

The results of this study confirm that depressed FM patients commonly experience both atypical and melancholic depressive features. In this study, atypical depression was 1.5 times more common than melancholic depression. Furthermore, while the melancholic group exhibit greater depression severity, those with atypical depression appear to have fibromyalgia symptoms an average of six years longer than those with melancholic depression. The presence of depression subtypes in depressed FM patients may have significant research and clinical implications. In research, a failure to account for depression subgroups may lead to regression towards the means in some studies of HPA function. Classic research provides evidence that the successful pharmacologic treatment of ADE differs from the treatment for MDE [51,52] and is proposed to be due to differing biological dysfunction of the HPA axis, locus ceruleus-noradrenergic system and the physiological stress systems. Thus, depression subtypes need to be addressed in future studies and clinical practice to eliminate the possibility of making erroneous conclusions from the data.

## Competing interests

The authors declare that they have no competing interests.

## Authors' contributions

RLR was involved in study design, acquisition of data, data analyses, interpretation of data, had full access to all data in this study and takes responsibility for the integrity of the data and accuracy of the analyses. RLR was the primary author of the manuscript and finalized it after comments from the other authors. KDJ participated in study design, interpretation of data and development and editing of the manuscript. RLW carried out the regression analyses and assisted in writing the odds ratio section. LJW commented on drafts of the manuscript. RMB constructed the FIQ, participated in study design and interpretation of data, made critical suggestions for the development of the manuscript as well as significant contributions to the final manuscript. All authors have read and approved the final manuscript.

## Pre-publication history

The pre-publication history for this paper can be accessed here:

http://www.biomedcentral.com/1471-2474/11/120/prepub

## Supplementary Material

Additional file 1 **DSM-IV-TR mood disorder episode specifiers for melancholic features and atypical features**. Lists the specific mood disorder diagnostic features for melancholic and atypical depression subtypes. Click here for file

Additional file 2 **Correlations among diagnostic subtype and atypical and melancholic episode specifiers using Spearman's Rho with p values**. Displays the correlation matrix for the diagnoses of ADE versus MDE with all five of the ADE-specific diagnostic features and all eight of the MDE-specific diagnostic features. Click here for file
