# Supplementary material for: Atypical depression is more common than melancholic in fibromyalgia: an observational cohort study
Source: BMC Musculoskelet Disord. 2010 Jun 14;11:120. doi: 10.1186/1471-2474-11-120 (PMC2909161; doi:10.1186/1471-2474-11-120)
Supplement: Additional file 1 — DSM-IV-TR mood disorder episode specifiers for melancholic features and atypical features. Lists the specific mood disorder diagnostic features for melancholic and atypical depression subtypes. [file 1471-2474-11-120-S1.PDF]

Melancholic Features Specifier

Specify if **With Melancholic Features** (can be applied to the current or most recent Major Depressive Episode in Major Depressive Disorder and to a Major Depressive Episode in Bipolar I or Bipolar II Disorder only if it is the most recent type of mood).

A. Either of the following, occurring during the most severe period of the current episode:

1. Either loss of pleasure in all, or almost all, activities
2. Lack of reactivity to usually pleasurable stimuli (does not feel much better, even temporarily, when something good happens.

B. Three or more of the following:

1. distinct quality of depressed mood (i.e., the depressed mood is experienced as distinctly different from the kind of feeling experienced after the death of a loved one)
2. depression regularly worse in the morning
3. early morning awakening (at least two hours before usual time of awakening)
4. marked psychomotor retardation or agitation
5. significant anorexia or weight loss
6. excessive or inappropriate guilt

Atypical Features Specifier

Specify if **With Atypical Features** (can be applied when these features predominate during the most recent 2 weeks of a current Major Depressive Episode in Major Depressive Disorder or in Bipolar I or Bipolar II Disorder when a current major Depressive Episode is the most recent type of mood episode, or when these feature predominate during the 2 years of Dysthymic Disorder; if the Major Depressive Episode is not current it applies if the feature predominates during any 2 week period).

A. Mood reactivity (i.e., mood brightens in response to actual or potential positive events)

B. Two (or more) of the following features:

1. significant weight gain or increase in appetite
2. hypersomnia
3. leaden paralysis (i.e., heavy leaden feelings in arms or legs)
4. long-standing pattern of interpersonal rejection sensitivity (not limited to episodes of mood disturbance) that results in significant social or occupational impairment

C. Criteria are not met for With Melancholic Features or With Catatonic Features during the same episode.
