# Supplementary material for: Atypical depression is more common than melancholic in fibromyalgia: an observational cohort study
Source: BMC Musculoskelet Disord. 2010 Jun 14;11:120. doi: 10.1186/1471-2474-11-120 (PMC2909161; doi:10.1186/1471-2474-11-120)
Supplement: Additional file 2 — Correlations among diagnostic subtype and atypical and melancholic episode specifiers using Spearman's Rho with p values. Displays the correlation matrix for the diagnoses of ADE versus MDE with all five of the ADE-specific diagnostic features and all eight of the MDE-specific diagnostic features. [file 1471-2474-11-120-S2.PDF]

	1	*2*	*3*	*4*	*5*	*6*	*7*	*8*	*9*	*10*	*11*	*12*	*13*	*14*	*15*	*16*
1. **MDE Diagnosis**	1															
2. Anhedonia	**.624*** **	1														
3. Lack of reactivity	**.446*** **	.093	1													
4. Distinct quality of depressed mood	**.258***	**.242***	.177	1												
5. Depression regularly worse in AM	**.300***	.174	.034	-.138	1											
6. Early morning awakening	**.467*** **	**.298***	.194	.179	.163	1										
7. Psychomotor retardation or agitation	**.372** **	.189	.197	.091	.100	.210	1									
8. Significant anorexia or weight loss	**.330** **	.042	.186	.146	.049	.212	.021	1								
9. Excessive or inappropriate guilt	**.449*** **	**.403*** **	.215	**.264***	.033	**.319** **	**.265***	.080	1							
10. **ADE Diagnosis**	**-1.0*** **	**-.624*** **	**-.446*** **	**-.258***	**-.300***	**-.467*** **	**-.372** **	**-.330** **	**-.449*** **	1						
11. Mood reactivity	**-.416*** **	**-.241***	**-.777*** **	-.107	-.183	-.172	-.162	-.222	-.092	**.416*** **	1					

12. Significant weight gain or inc. appetite	**-.300***	-.112	-.139	.033	.026	-.163	.080	**-.284***	.028	**.003***	.085	1				
13. Hypersomnia	-.179	-.212	-.134	-.035	-.167	-.111	-.005	-.121	-.071	.179	.215	-.099	1			
14. Leaden paralysis	-.150	.095	**-.334****	-.039	.111	-.098	-.129	.057	-.104	.150	-.042	.137	-.196	1		
15. Interpersonal rejection sensitivity	-.232	-.116	**-.278***	**-.252***	-.093	-.146	-.068	-.113	-.177	.232	.133	-.161	.091	.158	1	
16. Criteria not met for MDE	**.844*****	**.779*****	**.461*****	**.250***	.207	**.383*****	**.342****	.187	**.488*****	**-.844*****	**-429*****	**-.268***	-.161	-.155	**-.285***	1

Abbreviations: MDE, Melancholic depressive episode; ADE, atypical depressive episode; AM, morning; inc., increased.
